# Interventional Fairness with Indirect Knowledge of Unobserved Protected Attributes

**DOI:** 10.3390/e23121571

**Published:** 2021-11-25

**Authors:** Sainyam Galhotra, Karthikeyan Shanmugam, Prasanna Sattigeri, Kush R. Varshney

**Affiliations:** 1 Department of Computer Science, University of Chicago, Chicago, IL 60637, USA; 2 IBM Research, Yorktown Heights, NY 10598, USA; karthikeyan.shanmugam2@ibm.com (K.S.); psattig@us.ibm.com (P.S.); krvarshn@us.ibm.com (K.R.V.)

**Keywords:** causal fairness, responsible data science

## Abstract

The deployment of machine learning (ML) systems in applications with societal impact has motivated the study of fairness for marginalized groups. Often, the protected attribute is absent from the training dataset for legal reasons. However, datasets still contain proxy attributes that capture protected information and can inject unfairness in the ML model. Some deployed systems allow auditors, decision makers, or affected users to report issues or seek recourse by flagging individual samples. In this work, we examine such systems and consider a feedback-based framework where the protected attribute is unavailable and the flagged samples are indirect knowledge. The reported samples are used as guidance to identify the proxy attributes that are causally dependent on the (unknown) protected attribute. We work under the causal interventional fairness paradigm. Without requiring the underlying structural causal model a priori, we propose an approach that performs conditional independence tests on observed data to identify such proxy attributes. We theoretically prove the optimality of our algorithm, bound its complexity, and complement it with an empirical evaluation demonstrating its efficacy on various real-world and synthetic datasets.

## 1. Introduction

Due to the societal impact of automated systems, fairness in supervised learning has been a topic of prime importance. There have been numerous advances in defining fairness in terms of associational and causal effects of protected attributes on the prediction attribute [1,2,3,4], thereby mitigating unwanted bias. The majority of these algorithms assume that the protected attribute is accurately specified for the training dataset, which is then used to mitigate unwanted biases by processing the input dataset or modifying the training algorithm (in-processing) or post-processing the output of the prediction algorithm. However, the protected attribute is often unavailable or anonymized for legal reasons [5,6,7].

The absence of protected attributes from the training dataset does not guarantee fairness of the prediction algorithm. One of the primary reasons for this is the presence of proxy attributes that are causally dependent on the protected attributes. In such settings, a key challenge to ensure fairness is to identify these proxy attributes that may percolate bias into the prediction algorithm and then develop ways to mitigate such biases. Even if the dataset lacks any information about these attributes, software testing by legal auditors, recourse analysis of certain samples [8], or complaints from customers often uncover the presence of bias. In this work, we formalize a framework that leverages such indirect knowledge to identify proxy attributes, which can then help to improve fairness. We motivate this setting with the following example.

**Example** **1.** *Imagine that you are a manager examining a machine learning-powered resume screening app that your software company is starting to use internally [9]. You notice that a candidate named Latanya Sweeney—with an S.M. degree in electrical engineering and computer science from MIT and professional experience in minimizing privacy risk—has not been prioritized for your requisition for a staff software engineer to work on a HIPAA-compliant cloud infrastructure project. Suspecting algorithmic bias, you flag Latanya’s resume as feedback to the resume app.*

In this example of possible unfairness, neither the app nor the manager had access to any protected attributes such as race and gender for legal reasons [5,6]. The missingness of the protected attribute, however, did not prevent the manager from mentally using proxies for race and gender to flag the prediction. In this case, the name Latanya Sweeney is correlated with black women. If the machine learning model behind the app did have unwanted bias providing systematic disadvantage to black people and/or women, the algorithm must have used proxy attributes (like zip code, projects, or writing style) to reconstruct the information in the protected attributes. However, it is difficult to know what those proxy attributes were; it is usually not as simple as just the name of the individual or their zip code.

In this paper, we study fairness in terms of the causal effect of protected attributes on the prediction output/outcome attribute [1,2,3,4] and sought to identify the proxy attributes that are causally dependent on the protected attributes (that we do not know and do not have). A variable *X* is said to be causally dependent on another attribute $X^{\prime }$ if $X^{\prime }\to X$ in the causal graph, i.e., *X* is functionally dependent on $X^{\prime }$ and any manipulation of $X^{\prime }$ would impact *X*. However, we needed some extra information to help us on this quest. The information we utilized is precisely the indirect knowledge that we can glean from the flagging of possibly unfair decisions that the manager in our example submitted as feedback. We do not assume that the causal graph is known a priori.

We formalized the feedback-based framework to identify proxy attributes that are causally dependent on the unknown protected attribute. In terms of the causal graph, a proxy attribute is defined as the child of a protected attribute. We proposed efficient polynomial time algorithms that identify various connectivity properties of the causal graph that differ in the input dataset and the samples that are flagged by an auditor (indirect knowledge). It then uses these properties to identify constraints over pairs of input attributes, which are then used to formulate a constraint satisfaction problem (CSP). The solution of the CSP returns the set of proxy attributes.

**Contributions.** Our primary contributions are as follows.

1. We formalized a novel problem of using indirect signals to identify proxy attributes that are causally dependent on the protected attribute.
2. We identified unique connectivity properties of the causal graph, which are leveraged to develop a suite of efficient polynomial time algorithms that do not require the causal graph as an input. Our proposed techniques use off-the-shelf conditional independence tests to identify these attributes.
3. We proved theoretical guarantees that our algorithm accurately identifies the proxy attributes and runs in polynomial time. We showed that the complexity of our algorithm is linear in the number of attributes for sparse graphs.
4. We performed an end-to-end evaluation of our proposed techniques on various real-world and synthetic datasets. In real-world datasets, we showed that the classifier trained using our methods is fair and maintains high accuracy. On synthetic datasets, we validated the correctness of our algorithm by comparing with the ground truth.

## 2. Problem Setup

We denote random variables (also known as dataset attributes or features) by uppercase letters like X,S,A and their corresponding sample values in lowercase like x,s,a. Table 1 summarizes the notation.

**Causal DAG and interventions** A *causal* directed acyclic graph (DAG), *G* over a set of attributes V is a DAG that models the functional dependence between attributes in V. Each node *X* represents an attribute in V that is functionally determined by its parents Pa(X) in the DAG and some unobserved variables. An intervention to a causal graph is where an attribute *X* is set to some specific value, say *x*, and its effect on the distribution of the learned target attribute *Y* is observed. The do-operator allows this effect to be computed on a causal DAG, denoted P(Y|do(X=x)). To compute this value, we assumed that *X* is determined by a constant X=x. This assumption is equivalent to a modified graph with all incoming edges into *X* removed, and the value of *X* was set to *x*.

We assumed that the causal graph *G* on V is faithful to the observational distribution on V. This means that if two nodes *A* and *B* are connected by an edge in the causal graph, the data cannot result in any incorrect conditional independence of the form A⊥B∣C for any subset C⊂V\{A,B}. It is one of the most common assumptions in the causal discovery literature [1,3,10,11,12,13,14,15,16,17,18,19]. We use ⊥ to denote independence. We denote the edges of the causal graph *E* as a list of pairs $\left(X_{1},X_{2}\right)$ such that either $X_{1}$ causes $X_{2}$ or vice versa.

**Unobserved Protected Attribute** Consider a dataset *D* consisting of attributes $V=\{X_{1},\ldots$, $X_{n}\}$ along with a target attribute *Y*. Let *S* denote the protected attribute that is not available in the dataset *D*. *S* is considered as the common confounder for the set of attributes $V^{\prime }\subseteq V$. This is generally the case in settings where the protected attribute is the root node (has no parent) of the causal graph [3].
**Interventional Fairness** In this work, we consider the causal interventional fairness [3] paradigm that does not allow the protected attributes to affect the classifier output $Y^{\prime }$ through any attribute that is not admissible (A). Intuitively, an admissible attribute is the one that is allowed to percolate bias into the training algorithm. In Example 1, attributes like race and gender are considered protected attributes, and user preferences like type of job and expected salary are admissible.

**Definition** **1** (Causal Interventional Fairness). *For a given set of admissible attributes A, a classifier is considered fair if for any collection of values a of A and output $Y^{\prime }$, the following holds: $Pr\left(Y^{\prime }=y|do\left(S\right)=s,do\left(A=a\right)\right)=Pr\left(Y^{\prime }=y|do\left(S\right)=s^{\prime },do\left(A=a\right)\right)$ for all values of A, S and $Y^{\prime }$.*

Intuitively, this definition means that the probability distribution of the classifier output $Y^{\prime }$ is independent of the protected attributes when we intervene on the admissible attributes. In terms of the causal graph, this holds when all paths from the protected attribute to $Y^{\prime }$ are blocked by the admissible attributes. For more details about this definition, please refer to [3]. As discussed in the example, the current classifier output $Y^{\prime }$ does not satisfy this fairness criterion, and we wanted to identify the proxy attributes in order to train a fair classifier.

**Feedback Attribute** In this problem setup, we assume that a biased classifier outputs $Y^{\prime }$ are available and that an auditor inspects a subset of these records to identify biased outcomes. These flagged records are denoted with an extra attribute *F*, where F=1 denotes an example that was flagged by the auditor. As discussed in Example 1, the auditor processes a subset of the features, say, $V^{\prime }\subseteq V$, to flag a data point. Therefore, *F* is a function of a subset $V^{\prime }\subseteq V$ and the learned target $Y^{\prime }$ such that F=1 refers to a biased prediction. In terms of the causal graph, the attributes that were used as a signal to flag the classifier output are parents of *F*.
**Complaint set.** In order to define the complaint set, we assume a subset of the records from marginalized groups are discriminated, and a small subset of these discriminated records are reported as complaints. Therefore, all individuals in the complaint set are assumed to correspond to a specific subset of the marginalized group. The set of complaints are denoted by $D^{\prime }$, comprising attributes V for a small subset where F=1. (Note that the complaints $D^{\prime }$ does not contain all samples that suffer from biased prediction but only the ones that have been flagged.) Therefore, any conditional independence test of the form $A{⊥}_{D^{\prime }}B|C$ on the sample $D^{\prime }$ is equivalent to conditioning on the attribute *F* along with *C*, denoted by $\left(A{⊥}_{D}B|C,F\right)$. Whenever it is clear from context, we ignore the subscript *D* from the expressions. Unless specified, we always write the expression in terms of ${⊥}_{D}$. The operator ${⊥}_{D^{\prime }}$ is equivalent to ${⊥}_{D}$ with a conditioning on *F*. Since the feedback F=1 refers to a sample of biased predictions, we assumed that the majority of the samples with F=1 correspond to the members of marginalized or otherwise unprivileged communities.

**Assumption** **1.** *Considering the set of complaints (dataset $D^{\prime }$ where F=1), the protected attribute S=s is fixed for some records in the marginalized group S=s that have been flagged.*

This assumption is crucial to ensure that the feedback set $D^{\prime }$ contains indirect information about the marginalized group of individuals. Without this assumption, the set $D^{\prime }$ cannot be used to relate the complaints with the marginalized group. Note that the set $D^{\prime }$ does not contain all datapoints that have S=s. Therefore, adding a new column that treats all records in feedback set as S=s and all others as $S=s^{\prime }$ cannot be used as the protected attribute of individuals. Let $V_{F}\subseteq V$ denote the set of attributes that are used by the auditor to flag the datapoint. In terms of the causal graph, *F* is functionally dependent on *F*. Since *F* is a common descendant of all these attributes, any pair of attributes $X_{1},X_{2}\in V_{F}$ cannot be d-separated over $D^{\prime }$ i.e., (X1⊥D′X2|A)≡(X1⊥DX2|A,F),∀A⊆V\{X1,X2}.

**Proxy variables.** We defined the proxy variables as the non-admissible set of attributes that are functionally dependent on the unobserved protected attribute and that, therefore, have the maximum causal impact of the protected attribute. Due to the absence of the protected attribute, considering the proxy attributes as protected while employing any prior fairness-aware learning algorithm would guarantee a causally fair classifier. More formally, we claim the following.

**Lemma** **1.** *Consider a causal graph G over a set of attributes V, with unobserved protected attribute S. Let Children of the protected attribute S be denoted by Ch(S). If*

$$Pr\left(Y^{\prime }|do\left(Ch\left(S\right)\backslash A\right)=c,do\left(A\right)=a\right)=P\left(Y^{\prime }|do\left(Ch\left(S\right)\backslash A\right)=c^{\prime },do\left(A\right)=a\right)$$

$$\mathrm{then}Y^{\prime }\mathrm{is}\;\mathrm{causally}\;\mathrm{fair}\;i.e.,\;P\left(Y^{\prime }|do\left(A\right)=a,do\left(S\right)=s\right)=P\left(Y^{\prime }|do\left(A\right)=a,do\left(S\right)=s^{\prime }\right)$$

**Proof.**  Let T denote the children of *S* in the causal graph. If $Pr\left(Y^{\prime }|do\left(T\right)=c,do\left(A\right)=a\right)=P\left(Y^{\prime }|do\left(T\right)=c^{\prime },do\left(A\right)=a\right)$, then all paths from the attributes T to $Y^{\prime }$ are blocked when incoming edges of T and A are removed from *G*. In order to show that a classifier that obeys the condition of causal fairness with respect to *S*, we need to prove the following. After removing all incoming edges of *S* and A, there should be no directed paths from *S* to $Y^{\prime }$ without a collider ($Y^{\prime }$ should not be a descendant of *S*). Since all incoming edges of *S* have been removed, all directed paths from *S* to $Y^{\prime }$ pass through the children T. These paths $S\to X\to \ldots \to Y^{\prime }$ where X∈T: these paths that contain outgoing edges from T are all blocked because $Pr\left(Y^{\prime }|do\left(T\right)=c,do\left(A\right)=a\right)=P\left(Y^{\prime }|do\left(T\right)=c^{\prime },\left(A\right)=a\right)$.

This shows that whenever the proxy variables are considered as protected while training a fair classifier, causal fairness of the outcome is guaranteed.    □

Note that any superset of the children of *S* (multi-hop descendants) is a valid set of proxy variables as they may be causally dependent on *S*. However, Children(S) is the smallest set of attributes that need to be accounted for fair classification. Considering more variables as proxies could affect the overall classification accuracy.

## 3. Problem Statement and Solution Approach

In this section, we first define the problem statement and give high-level observations about the connectivity properties of the causal graph. We then use these properties to design a simple algorithm, which is then improved by formulating a constraint satisfaction problem. We then improve the efficiency of the algorithm by leveraging the sparsity properties of causal graphs.

Based on the notation we defined in the previous section, we can state the problem of identifying proxy-protected attributes as follows.

**Problem** **1.** *Given a dataset D comprising attributes V with a classifier output $Y^{\prime }$ and a biased feedback set $D^{\prime }$, identify the smallest subset $V^{\prime }\subseteq V$ such that the hidden protected attribute S is a common confounder for the attributes in $V^{\prime }$.*

**Figure 1:**
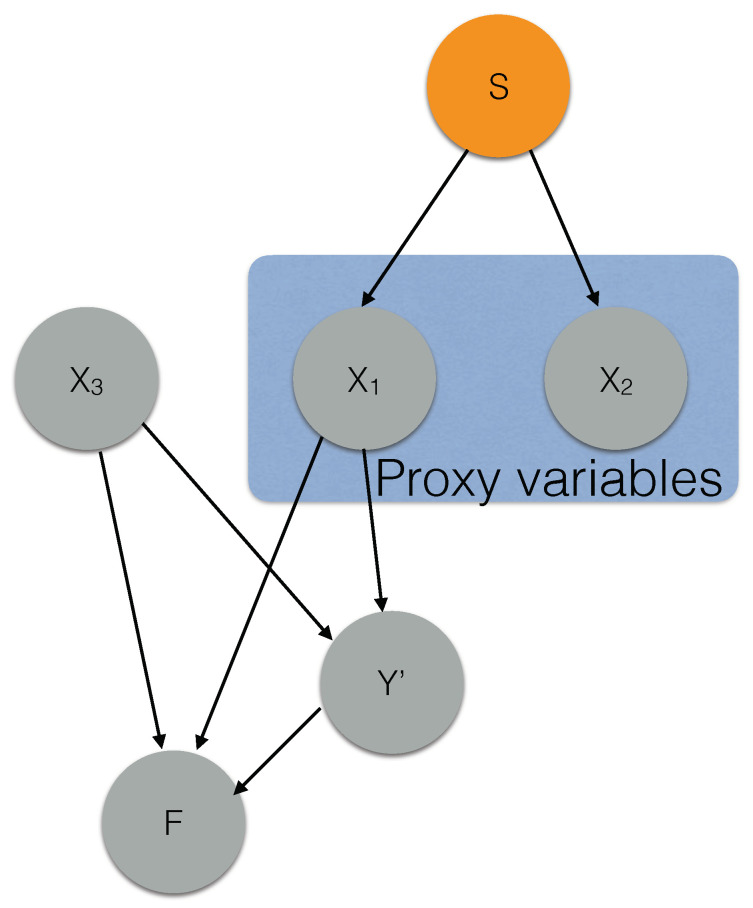
Example dataset where the protected attribute *S* and the causal graph are unobserved. The attribute $Y^{\prime }$ denotes the learned target attribute; *F* is the feedback attribute, which refers to the selection variable for the complaints flagged by an auditor; and $X_{1}$ and $X_{2}$ are proxy attributes.

Now let us work towards a solution. Let us first identify the condition under which proxies for the protected attribute can be identified from observational data and develop efficient techniques for the same. Consider a simple toy causal graph example, shown in Figure 1, where only the protected attribute is unobserved. We made a simplistic assumption that only the protected attribute is unobserved for this example. Our technique and theoretical analysis extends to the general case where many other attributes may be unobserved. Note that we have access to the training dataset *D* containing $V=\{X_{1},X_{2},X_{3}\}$ and a small feedback dataset $D^{\prime }$, which is equivalent to conditioning F=1. The subset of the data that has F=1 may not overlap with the training data. In this example, the attributes that impact *F* are $V_{F}=\left\{X_{1},X_{3}\right\}$, and the proxy attributes are $V^{\prime }=\left\{X_{1},X_{2}\right\}$. We can see that identifying proxy attributes is an easy task if the causal graph is known. Now, let us look at some of the properties of *D* and $D^{\prime }$ that can help in the absence of the causal graph.

1. Consider the attributes $X_{1}$ and $X_{2}$, which are confounded by the protected attribute *S* and $(X_{1},X_{2})\notin E$. Since *S* is unobserved in the dataset *D*, $X_{1}$ and $X_{2}$ cannot be d-separated, i.e., X1⊥DX2|A,∀A⊆V\{X1,X2}. However, the feedback *F* is equivalent to considering a smaller sub-population (conditioning on *S*), which breaks the confounding relation between $X_{1}$ and $X_{2}$. Therefore, $X_{1}{⊥}_{D}X_{2}|F\equiv X_{1}{⊥}_{D^{\prime }}X_{2}$. This equation can be easily tested by performing a CI test on the flagged samples.
2. Consider the attributes $X_{1}$ and $X_{3}$, which are not confounded by the protected attribute *S*. For such attributes, there exists a subset $A\subseteq V\backslash \{X_{1},X_{3}\}$ such that $X_{1}⊥X_{3}|A$. In Figure 1, A=ϕ. However, $X_{1},X_{3}\in V_{F}$ means that the collider path $X_{1}\to Y^{\prime }\leftarrow X_{3}$ gets unblocked given *F*, implying X1⊥DX3|A,F≡X1⊥D′X3|A, $\forall A\subseteq V\backslash \{X_{1},X_{3}\}$. Therefore, $X_{1}$ and $X_{3}$ can never be d-separated in the feedback dataset $D^{\prime }$.

These observations show that different attributes in the causal graph satisfy different properties based on their membership. We formalize these intuitions for general graphs and prove the following properties for any pair of attributes. Lemma 2 proves the condition in which $X_{1}$ and $X_{2}$ can be d-separated with respect to *D* and $D^{\prime }$, if $X_{1},X_{2}$ are proxy attributes.

**Lemma** **2.** *Consider a pair of attributes $X_{1}$ and $X_{2}\in V$ with $(X_{1},X_{2})\notin E$. $X_{1},X_{2}\in V^{\prime }$, and at least one of $X_{1}$ and $X_{2}$ does not belong to $V_{F}$ iff*

1. *X1⊥X2|A for all $A\subseteq V\backslash \{X_{1},X_{2}\}$ and*
2. *$X_{1}⊥X_{2}|A,F$ for some $A\subseteq V\backslash \{X_{1},X_{2}\}$*

**Proof.**  We consider the two sides of the lemma separately. First, let us assume that $(X_{1},X_{2})\notin E$, $X_{1},X_{2}\in V^{\prime }$ and at least one of $X_{1}$ and $X_{2}$ do not belong to $V_{F}$. This implies the following conditions.

- If $X_{1},X_{2}\in V^{\prime }$, then *S* is a common confounder for both $X_{1}$ and $X_{2}$. Therefore, $X_{1}$ and $X_{2}$ can not be d-separated, implying (X1⊥X2|A)∀A⊆V\{X1,X2} because *S* is not observed.
- If at least one of $X_{1}$ and $X_{2}$ do not belong to $V_{F}$ and $(X_{1},X_{2})\notin E$, then there exists some *A* such that $X_{1}$ and $X_{2}$ are d-separated given A,F. This is because conditioning on the feedback *F* implies S=1 (conditioning on *S*), which breaks the confounding relationship between $X_{1}$ and $X_{2}$.

For the other direction,

- If $X_{1}⊥X_{2}|A,F$ for some $A\subseteq V\backslash \{X_{1},X_{2}\}$, then both $X_{1}$ and $X_{2}$ cannot be in $V_{F}$ and $(X_{1},X_{2})\notin E$. This is because if $X_{1},X_{2}\in V_{F}$, then X1⊥X2|A,F for any *A* (by definition of $V_{F}$).
- If X1⊥X2|A for all *A* but $\exists A^{\prime }|X_{1}⊥X_{2}|A^{\prime },F$ (we also know that $(X_{1},X_{2})\notin E$.). Suppose $X_{1},X_{2}$ are not confounded by *S*. Conditioning on *F* and $A^{\prime }$ blocks all paths from $X_{1}$ to $X_{2}$. Since conditioning on *F* does not open any new paths between $X_{1}$ and $X_{2}$, there will exist $A^{\prime }$ such that $X_{1}⊥X_{2}|A^{\prime }$ if $X_{1}$ and $X_{2}$ are not confounded by *S*. This is a contradiction, implying $X_{1}$ and $X_{2}$ are confounded by *S*.

   □

Lemma 3 proves the properties for $X_{1}$ and $X_{2}$, whenever both of these attributes are considered by the auditor to flag the datapoint.

**Lemma** **3.** *For a pair of attributes $X_{1}$ and $X_{2}\in V$ with $(X_{1},X_{2})\notin E$, $X_{1},X_{2}\in V_{F}$, and at least one of $X_{1}$ and $X_{2}$ does not belong to $V^{\prime }$ iff*

1. *$X_{1}⊥X_{2}|A$ for some $A\subseteq V\backslash \{X_{1},X_{2}\}$*
2. *X1⊥X2|A,F for all $A\subseteq V\backslash \{X_{1},X_{2}\}$*

**Proof.**  First, let us assume that $(X_{1},X_{2})\notin E$, $X_{1},X_{2}\in V_{F}$, and at least one of $X_{1}$ and $X_{2}$ do not belong to $V^{\prime }$.

- If at least one of $X_{1}$ and $X_{2}$ do not belong to $V^{\prime }$ and $(X_{1},X_{2})\notin E$, then there exists some $A\subseteq V\backslash \{X_{1},X_{2}\}$ such that $X_{1}$ and $X_{2}$ are d-separated given *A*.
- If $X_{1},X_{2}\in V_{F}$, then $X_{1}\to F\leftarrow X_{2}$ forms a collider path, which is unblocked given *F*. Therefore, (X1⊥X2|A,F)∀A⊆V\{X1,X2}

For the other direction,

- If $X_{1}⊥X_{2}|A$ for some $A\subseteq V\backslash \{X_{1},X_{2}\}$, then both $X_{1}$ and $X_{2}$ cannot be in $V^{\prime }$ and $(X_{1},X_{2})\notin E$. This is because if $X_{1},X_{2}\in V^{\prime }$, then X1⊥X2|A,∀A⊆V because of an unblocked path $X_{1}\leftarrow S\to X_{2}$
- If X1⊥X2|A,F for all *A* but ∃A such that $X_{1}⊥X_{2}|A$. We also know that $(X_{1},X_{2})\notin E$. Consider the *A* for which $X_{1}⊥X_{2}|A$. In this causal graph, all paths from $X_{1}$ to $X_{2}$ are blocked but on conditioning *F* along with *A*, some path gets unblocked. Since $X_{1}$ and $X_{2}$ cannot be d-separated when we condition on *F*, $X_{1},X_{2}\in V_{F}$.

   □

For simplicity, we proved these properties for two cases. These properties can be extended for any combination of attributes based on their occurrence in $V^{\prime }$ and $V_{F}$. Table 2 lists these conditional independence/dependence behavior of all possible combination of attributes $X_{1}$ and $X_{2}$. For example, the first row shows that if $X_{1}$ and $X_{2}\in V_{F}\cap V^{\prime }$, then X1⊥X2|A for all $A\subseteq V\backslash \{X_{1},X_{2}\}$.

### 3.1. Simple Algorithm

Using the properties listed in Table 2, Algorithm 1 presents the pseudocode of a simple algorithm that identifies proxy-protected attributes. It iterates over all pair of attributes and performs two types of conditional independence tests (one with conditioning on $A\subseteq V\backslash \{X_{1},X_{2}\}$ and the other with conditioning on *A* and *F*, i.e., with respect to $D^{\prime }$). Following Lemma 2, if ∃A such that $X_{1}⊥X_{2}|F,A$ and X1⊥X2|A,∀A, then $X_{1}$ and $X_{2}$ are both added to the set $V^{\prime }$. Lemma 4 analyzes the conditions when an attribute $X_{1}\in V^{\prime }$ is correctly identified by Algorithm 1.
**Algorithm 1** Proxy identification.1:**Input:** attributes V,F2:$V^{\prime }\leftarrow \phi$3:**for** $X_{1}\in V\backslash V^{\prime }$ **do**4:   **for** $X_{2}\in V$ **do**5:     **if** $\exists A\subseteq V\backslash \left\{X_{1},X_{2}\right\}|\left(X_{1}⊥X_{2}|F,A\right)$ **then**6:        **if** ∀A⊆V\{X1,X2}∣(X1⊥X2|A) **then**7:          $V^{\prime }\leftarrow V^{\prime }\cup \left\{X_{1},X_{2}\right\}$8:**return** $V^{\prime }$

**Lemma** **4.** *An attribute $X\in V^{\prime }$ is correctly identified to belong to $V^{\prime }$ if $\exists X^{\prime }\in V^{\prime }$ such that $(X,X^{\prime })\notin E$ and $|V_{F}\cap \left\{X,X^{\prime }\right\}|\leq 1$.*

**Proof.**  Consider an attribute $X\in V^{\prime }$, and let $X^{\prime }\in V^{\prime }$ such that $V_{F}\cap \left\{X,X^{\prime }\right\}\leq 1$. Therefore, one of *X* and $X^{\prime }\notin V_{F}$. Using Lemma 2, X⊥X′|A, $\forall A\subseteq V\backslash \{X_{1},X_{2}\}$, and $\exists A\subseteq V\backslash \{X_{1},X_{2}\}$ such that $X⊥X^{\prime }|A,F$ holds. Therefore, Algorithm 1 correctly identifies *X* and $X^{\prime }\in V^{\prime }$.    □

However, Algorithm 1 has two main drawbacks:

1. In dense graphs, there may exist an attribute $X\in V^{\prime }$ such that $∄X^{\prime }\in V^{\prime }$ where $(X,X^{\prime })\notin E$. Such attributes may not be identified by Algorithm 1.
2. The conditional independence test of the form X1⊥X2|A,∀A⊆V\{X1,X2} requires us to test the conditional dependence for every subset $A\subseteq V\backslash \{X_{1},X_{2}\}$. This condition requires an exponential number of conditional independence tests.

We now present a constraint satisfaction problem-based formulation that overcomes the first limitation (Section 3.2) and an efficient mechanism to optimize the total number of required conditional independence tests (Section 3.3).

### 3.2. Constraint Satisfaction Formulation

In this section, we leverage the properties of Table 2 to formulate a constraint satisfaction problem (CSP), which is then solved to identify the membership of the attributes. Let us first define the set of variables for this CSP. For each attribute X∈V, define two binary variables $X^{F}$ and $X^{S}\in \left\{0,1\right\}$ such that $X^{F}=1$ if $X\in V_{F}$ and 0 otherwise. Similarly, $X^{S}=1$ if $X\in V^{\prime }$ and 0 otherwise. Given a pair of attributes $X_{1}$ and $X_{2}$, we can perform conditional independence tests as described in Table 2 and introduce one of the following constraints based on their output.

- If X1⊥X2|A,∀A⊆V\{X1,X2} and $\exists A\subseteq V\backslash \{X_{1},X_{2}\}$ such that $X_{1}⊥X_{2}|A,F$, then both $X_{1}$ and $X_{2}\in V^{\prime }$ and at least one of the two attributes does not belong to $V_{F}$ (Using Lemma 2). Therefore, $X_{1}^{S}=X_{2}^{S}=1$ and $X_{1}^{F}+X_{2}^{F}\leq 1$.
- If $\exists A\subseteq V\backslash \{X_{1},X_{2}\}$ such that $X_{1}⊥X_{2}|A$ and X1⊥X2|A,F$\forall A\subseteq V\backslash \{X_{1},X_{2}\}$, then both attributes $X_{1}$ and $X_{2}$ belong to $V_{F}$, and at least one of the attributes does not belong to $V^{\prime }$ (Using Lemma 3). Therefore, $X_{1}^{F}=X_{2}^{F}=1$ and $X_{1}^{S}+X_{2}^{S}\leq 1$
- If $\exists A\subseteq V\backslash \{X_{1},X_{2}\}$ such that $X_{1}⊥X_{2}|A$ and $\exists A^{\prime }\subseteq V\backslash \left\{X_{1},X_{2}\right\}$ such that $X_{1}⊥X_{2}|A^{\prime },F$, then $X_{1}$ and $X_{2}\notin V^{\prime }\cap V_{F}$. Therefore, $X_{1}^{F}+X_{1}^{S}+X_{2}^{F}+X_{2}^{S}\leq 2$.

Using this strategy, we introduce constraints for every pair of attributes $X_{1},X_{2}\in V$. The membership of all attributes can be identified by solving this constraint satisfaction problem. To solve this constraint satisfaction problem (containing at most O(n2) constraints), we can use any standard CSP solver [20]. Note that most of the presented constraints are binary, and we can easily implement a polynomial time solver to calculate their membership. An efficient implementation of this instance would be to construct a complete graph over the attributes V with constraints on nodes and edges. For example, the constraint of the form $X_{1}^{S}+X_{1}^{F}\leq 1$ is a constraint on the node (as these constraints involve a single attribute), and the ones of the form $X_{1}^{F}+X_{2}^{F}\leq 1$ refer to edge constraints. To identify a feasible solution, we iteratively remove the constraints by processing node constraints that fix the values of variables and then propagating their effect on the edge constraints. In this constraint satisfaction formulation, membership of all variables that have a unique value are correctly identified. All other variables that do not have a unique value cannot be classified correctly and are considered as proxy attributes. However, we next show that membership of all attributes are correctly identified for realistic settings (sparse graphs). The membership may not be identified in case a number of attributes have a very high degree (see Lemma 4). As an extreme case, membership of an attribute that is functionally dependent on all other attributes would not be identified by the CSP. However, it is impossible to identify its membership as all attributes are dependent on this high-degree attribute.

The main advantage of this algorithm over Algorithm 1 is that we leveraged properties from Table 2 to identify the membership of an attribute *X*. If an attribute *X* is attached to every other attribute $X^{\prime }\in V$, then our techniques would not be able to pin-point whether *X* is a proxy attribute or not. In such cases, it returns three sets of attributes (a) proxy attributes having $X^{S}=1$, (b) non-proxy attributes ($X^{S}=0$), and (c) undecided attributes (high-degree nodes for which $X^{S}$ is not uniquely determined). If all the proxy and undecided attributes are not used, the trained classifier is guaranteed to be fair.

### 3.3. Efficient Implementation

Algorithm 1 and the constraint satisfaction problem rely on conditional independence tests that consider all possible subsets $A\subseteq V\backslash \{X_{1},X_{2}\}$. Therefore, a naive implementation of Algorithm 1 requires $O(2^{\left|V\right|})$ tests. This may not be feasible for large values of |V|, especially when it has to be performed for all pairs of attributes.

In order to improve the overall complexity, we made the following observation for sparse causal graphs. If there exist two attributes $X_{1}$ and $X_{2}\notin V^{\prime }$ where $(X_{1},X_{2})\notin E$, then they are not connected to any length-2 collider path (paths of the form $X_{1}\to X^{\prime }\leftarrow X_{2}$ for some $X^{\prime }\in V$) iff $X_{1}⊥X_{2}|V\backslash \left\{X_{1},X_{2}\right\}$. This holds because when we condition on all attributes except $X_{1}$ and $X_{2}$, all paths from $X_{1}$ and $X_{2}$ are blocked except length-2 collider paths of the form $X_{1}\to X_{3}\leftarrow X_{2}$. Since there are no such paths, it means that the test X1⊥X2|A,∀A⊆V\{X1,X2} is equivalent to testing for X1⊥X2|V\{X1,X2} for such pairs of attributes. Lemma 5 extends this observation to general scenarios where the number of such length-2 collider paths between a pair of attributes is bounded.

**Lemma** **5.** *Consider a pair $X_{1}$ and $X_{2}$ such that $(X_{1},X_{2})\notin E$ and at least one of the two attributes does not belong to $V^{\prime }$. The following conditions hold:*

1. *$X_{1}$ and $X_{2}$ are independent when conditioned on all other attributes ($X_{1}⊥X_{2}|V\backslash \left\{X_{1},X_{2}\right\}$) iff there does not exist $X^{\prime }\in V$ such that $X_{1}\to X^{\prime }\leftarrow X_{2}$ form a collider path.*
2. *$\exists V_{1}$ such that $X_{1}⊥X_{2}|V_{1}$ where $|V_{1}|\geq n-t$ iff the number of attributes in set $V^{\prime }$ is less than t, where $V^{\prime }$ contains all attributes X∈V that form a length-2 collider path $X_{1}\to X\leftarrow X_{2}$ or X is a descendant of some attribute $X^{\prime }\in V^{\prime }$, where $X^{\prime }$ forms a length-2 collider path.*

**Proof of Lemma 5.**  Consider a pair of attributes $X_{1}$ and $X_{2}$ such that $(X_{1},X_{2})\notin E$ and at least one of $X_{1},X_{2}\notin V^{\prime }$. If $X_{1}$ and $X_{2}$ do not have any length-2 collider path, conditioning on all attributes d-separates $X_{1}$ and $X_{2}$. This holds because for any collider path of length more than 2 (say $X_{1}\to X_{i}\ldots \leftarrow X_{j}\leftarrow X_{2}$), then both $X_{i}$ or $X_{j}$ are conditioned. Similarly for any path with incoming edges into $X_{1}$ or $X_{2}$ (backdoor paths), the parents of both attributes are also conditioned on. Therefore, $X_{1}⊥X_{2}|V\backslash \left\{X_{1},X_{2}\right\}$.

If a set of attributes $X^{\prime },\left|X^{\prime }\right|\leq t$ where $X^{\prime }$ contains all *X* such that attributes forming length-2 collider of the form $X_{1}\to X\leftarrow X_{2}$ or *X* is a descendant of an attribute $X^{\prime }\in X^{\prime }$. In this case, $X_{1}$ and $X_{2}$ can be d-separated by conditioning on all attributes except $X^{\prime }$ because conditioning on any ancestor of $X_{1}$ and $X_{2}$ does not open new paths. Similarly, if the collider path has a length greater than 2, then the path is blocked by conditioning on all attributes that are not in $X^{\prime }$. For example, if the collider path is length 3, $X_{1}\to X_{3}\to X_{4}\leftarrow X_{2}$, then conditioning on $X_{3}$ and $X_{4}$ does not open this collider path.

More formally, consider any collider path of length greater than 2, say $X_{1}\to X_{i}\ldots X_{j}\leftarrow X_{2}$. If $X_{i},X_{j}\in X^{\prime }$, then all descendants of $X_{i}$ and $X_{j}$ also belong to $X^{\prime }$. Therefore, this path is blocked. If $X_{i}\notin X^{\prime }$, this path is blocked by conditioning on $X_{i}$, and conditioning on $X_{i}$ does not open any length-2 collider paths because $X_{i}\notin X^{\prime }$. Any >2 length collider path that is unblocked by conditioning on $X_{i}$ get blocked by another $X_{j^{\prime }}$, which is a child of $X_{1}$ or $X_{2}$ in that path. Therefore, conditioning on $V\backslash X^{\prime }$ does not open any path from $X_{1}$ to $X_{2}$.    □

Algorithm 2 uses this property to optimize the number of conditional independence tests required to calculate the membership of each attribute. It initializes with t=|V| (line 3) and iteratively decreases *t* to consider attributes with at most |V|−t length-2 collider paths. For an iteration *t*, it considers all subsets of V of size n−t (denoted by T) as the conditioning set (line 6). Using this conditioning set, it evaluates conditional independence constraints for every pair of attributes $X_{1},X_{2}\in V$ (Algorithm 3). These constraints are the same as the ones discussed in Section 3.2. The SolveCSP subroutine then solves the CSP with new constraints and removes the attributes from *U* for which $X^{S}$ has been uniquely determined (line 9). The procedure stops as soon as the $X^{S}$ values of all attributes X∈V have been uniquely identified (U=ϕ) and returns the subset for which $X^{S}=\left\{1\right\}$.
**Algorithm 2** Proxy identification.1:**Input:** attributes V,F2:U←V, C←ϕ3:$X^{S},X^{F}\leftarrow \left\{0,1\right\},\forall X\in V$4:t←|V|5:**while**t≥0 and U≠ϕ **do**6:   T← IdentifySubset (V,t)7:   C←C∪PairwiseConstraints(V,T)8:   SolveCSP(V,C)9:   $U\leftarrow \{X:0,1\in X^{S},X\in V\}$10:   t←t−111:$V^{\prime }\leftarrow \left\{X:X^{S}=\left\{1\right\}\right\}$12:**return** $V^{\prime }$
**Algorithm 3**Pairwise constraints.**Input:** Attributes V,F,TC←ϕ**for** $(X_{1},X_{2})\in V\times V$ **do**   **if** $\exists T\in T|X_{1}⊥X_{2}|T\backslash \left\{X_{1},X_{2}\right\}$ and X1⊥X2|T\{X1,X2},F∀T∈T **then**     $C\leftarrow C\cup \{X_{1}^{F},X_{2}^{F}\leftarrow 1\}$     $C\leftarrow C\cup \{X_{1}^{S}+X_{2}^{S}\leq 1\}$   **if** X1⊥X2∣T\{X1,X2} and $X_{1}⊥X_{2}|T\backslash \left\{X_{1},X_{2}\right\},F$ **then**     $C\leftarrow C\cup \{X_{1}^{S},X_{2}^{S}\leftarrow 1\}$     $C\leftarrow C\cup \{X_{1}^{F}+X_{2}^{F}\leq 1\}$   **if** $X_{1}⊥X_{2}|T\backslash \left\{X_{1},X_{2}\right\}$ and $X_{1}⊥X_{2}|T\backslash \left\{X_{1},X_{2}\right\},F$ **then**     $C\leftarrow C\cup \{X_{1}^{S}+X_{1}^{F}+X_{2}^{S}+X_{2}^{F}\leq 2\}$**return***C*
**PairwiseConstraints****.** Algorithm 3 presents the pseudocode for this subroutine. It iterates over pairs of attributes and performs CI tests to identify the corresponding constraint, guided by Table 2.

In order to prove the correctness of Algorithm 2, we argue that it does not introduce any spurious constraints in the CSP optimization. Lemma 6 shows that if a pair $X_{1}$ and $X_{2}$ have more than α length-2 collider paths, then $X_{1}$ and $X_{2}$ cannot be d-separated by conditioning on any subset of size more than n−α. Since each new constraint introduced by Algorithm 3 requires conditional independence of $X_{1}$ and $X_{2}$ with respect to some subset on *D* or $D^{\prime }$, it does not identify incorrect constraints. We now prove Lemma 6.

**Lemma** **6.** *Consider a pair of attributes $X_{1}$ and $X_{2}$ such that the total number of length-2 collider paths ($X_{1}\to X\leftarrow X_{2}$ where $X\in V^{\prime }$) is at least α. Any CI test between $X_{1}$ and $X_{2}$ conditioning on A where |A|>n−α returns X1⊥X2|A.*

**Proof.**  If a pair of attributes $X_{1}$ and $X_{2}$ have more than α length-2 collider paths, then conditioning on any subset of size more than n−α implies conditioning on at least one of the collider nodes. Therefore, X1⊥X2|A whenever |A|>n−α.    □

### 3.4. Time Complexity

We now analyze the running time of Algorithm 2 for commonly studied causal graph models. Theorem 1 bounds the total number of CI tests required for a degree-bounded graph, and then we extend our analysis to Erdos-Renyi graphs.

**Theorem** **1.** *For a causal graph where each node X∈V has a degree less than α and $|V^{\prime }\backslash V_{F}|>\alpha^{2}$, Algorithm 2 requires $O(n^{2})$ CI tests to identify all proxy attributes.*

**Proof.**  For a node *X* with degree <α, the maximum number of 2-hop neighbors of *X* is $\leq {\left(\alpha -1\right)}^{2}$. This analysis considers all edges as undirected and can be tightened by considering directions and splitting α into incoming and outgoing degrees of each node. Therefore, *X* can have at most ${\left(\alpha -1\right)}^{2}$ length-2 collider paths. This means that if $V^{\prime }\backslash V_{F}$ contains more than ${\left(\alpha -1\right)}^{2}$ two-hop and α−1 one-hop attributes, then $\exists X^{\prime }\in V^{\prime }$ such that $X^{\prime }$ is at least 2-hops away from *X*. Since $\alpha^{2}>{\left(\alpha -1\right)}^{2}+\left(\alpha -1\right)$, $\exists X^{\prime }\in V^{\prime }$ that satisfies this condition. Such attributes are identified in the CI test $X⊥X^{\prime }|F,V\backslash \left\{X,X^{\prime }\right\}$. Therefore, all attributes are correctly identified in 1 test for every pair of attributes.    □

**Erdős-Renyi Graphs.** We consider a randomized generative model for the causal graph construction where each pair of attributes are causally related independently with a probability *p*. We show that whenever $p<1/\sqrt{n}$, Algorithm 2 identifies all proxy attributes in $O(n^{2})$ running time. Such connectivity models for causal graphs have been widely studied [21]. Lemma 7 bounds the expected number of length-2 collider paths between a pair of attributes $X_{1}$ and $X_{2}$.

**Lemma** **7.** *Consider a pair of attributes $X_{1}$ and $X_{2}$ such that $(X_{1},X_{2})\notin E$. The probability that $X_{1}$ and $X_{2}$ have a length-2 collider path between them is less than $p^{2}\left(n-2\right)$.*

**Proof.**  Let $X_{v}$ denote a binary random variable such that $X_{v}=1$ if $X_{1}\to X\leftarrow X_{2}$ forms a collider path for X∈V. The probability that $(X_{1},X)\in E$ and $(X,X_{2})\in E$ is $p\times p=p^{2}$. Therefore, $Pr\left[X_{v}=1\right]=p^{2}$.    □

Using this result, we prove the following complexity of our algorithm.

**Theorem** **2.** *Algorithm 2 identifies the proxy attributes in less than $O(n^{2})$ CI tests if $p=o(\sqrt{1/n})$*

**Proof.**  Given a pair of attributes $X_{1}$ and $X_{2}$, the probability that $X_{1}$ and $X_{2}$ are within 2-hops from each other is $p^{2}\left(n-2\right)=o\left(1\right)$ if $p=o(\sqrt{1/n})$. Therefore, $\forall X\in V^{\prime }$, there will exist $X^{\prime }\in V^{\prime }$ such that $(X,X^{\prime })\notin E$ and the two attributes are more than 2-hops away. Therefore, X⊥X′|A∀A⊆V\{X,X′} and $X⊥X^{\prime }|A,F$ for some $A\subseteq V\backslash \{X,X^{\prime }\}$.

This means that all attributes in $V^{\prime }$ have been recovered in the first iteration of Algorithm 2.    □

### 3.5. Graphical Lasso-Based Algorithm

In this section, we study a specific class of causal graphs where the structural equations are Gaussian. In this setting, we show that Algorithm 2 can be implemented efficiently using the graphical lasso algorithm.

Graphical lasso [22] is one of the widely studied methods to infer the precision matrix of the underlying causal model in settings where the structural equations are Gaussian. (The precision matrix is the inverse of the covariance matrix; its non-zero values encode the edges in the graph.) Following the properties of Lemma 2, we know that X1⊥X2|A, $\forall A\subseteq V\backslash \{X_{1},X_{2}\}$ if $X_{1},X_{2}\in V^{\prime }$. Therefore the precision matrix identified over *D* would contain $\left(X_{1},X_{2}\right)$ as an edge. Similarly, Lemma 2 also shows that $\exists A\subseteq V\backslash \{X_{1},X_{2}\}$ such that $X_{1}⊥X_{2}|A,F$ iff $X_{1},X_{2}\in V^{\prime }$. This means that the entry corresponding $\left(X_{1},X_{2}\right)$ in the precision matrix will be 0. Using this property, a simple algorithm to identify the proxy attributes is as follows. (a) Step 1: Run graphical lasso on the original dataset *D*. Let *P* denote the returned precision matrix. (b) Step 2: Run graphical lass on the dataset $D^{\prime }$. Let $P^{\prime }$ denote the returned precision matrix. (c) Step 3: Calculate the set difference $P\backslash P^{\prime }$. All attributes with degree more than 0 in $P\backslash P^{\prime }$ are the proxy attributes. One of the advantages of this technique is that the graphical lasso algorithm is highly efficient, but it is restricted to multivariate Gaussian causal models and does not generalize to general datasets.

## 4. Experiments

In this section, we evaluate the effectiveness of our techniques to identify proxy attributes that capture protected information such that removing these attributes improves classifier fairness. The protected attributes are hidden from the dataset and are used only to evaluate the fairness of the learned classifier.

### 4.1. Setup

#### 4.1.1. Datasets

We consider the following real-world datasets.

- *Medical Expenditure* (MEPS) [23]: This dataset is used to predict the total number of hospital visits from patient medical information. Healthcare utilization is sometimes used as a proxy for allocating preventative care management. We consider “arthritis diagnosis” as admissible. Race is considered protected and is hidden for experimentation. The dataset contains 7915 training and 3100 test records.
- *German Credit* [24] dataset contains attributes of various applicants, and the goal was to classify them based on credit risk. The account status is taken as admissible, and whether the person is below the mean age is considered protected. The dataset contains 800 training and 200 test records.
- *Adult* dataset [25] contains demographic information of individuals along with their information on their level of education, occupation, working hours, etc. The task was to predict whether or not the annual income of an individual exceeds 50K. Race was treated as the protected attribute, and education was treated as admissible. The dataset contains around 32K training and 16K test records.

#### 4.1.2. Baselines

Our experimental setup is similar to that of [3], where the input dataset contains admissible attributes (denoted by A), referring to the set of attributes that are allowed to inject bias into the trained classifier. In the implementation of our algorithm, we identified all proxy attributes and trained a new classifier after removing them from the dataset. Due to the small size of A, classifiers trained on A tend to predict a single class if the training data are not balanced. Therefore, we compare the performance of the trained classifier on both original and balanced data. All algorithms were implemented in Python, and we use Scikit-Learn’s logistic regression classifier with default parameters.

Since causal fairness cannot be tested on real datasets, we evaluate the fairness of the classifier in terms of absolute odds difference (AOD) as a proxy. AOD is calculated as the difference in the false-positive rate and the true-positive rate between the privileged and unprivileged/marginalized groups. The set of privileged and unprivileged/marginalized groups are identified according to the sensitive attribute. For example, white individuals are considered privileged in MEPS dataset. The feedback sample is constructed randomly by considering a small sample of unprivileged records that received negative outcomes (less than 100 data points). We used the RCIT package [26] for CI testing, and the Glass package [27] for graphical lasso. These packages are in R. Unless specified, we used Algorithm 2 for our experiments. We considered the following baselines. (i) A uses the attributes in the admissible set. (ii) ALL uses all attributes present in the dataset.

### 4.2. Solution Quality

Table 3 compares the accuracy and average precision of the trained classifier along with absolute odds difference to measure fairness. Among all datasets, the accuracy of our approach is similar to All, and the fairness is similar to that of A. This experiment validates that the removal of proxy attributes from the dataset does not worsen the overall accuracy but helps to improve fairness of the trained classifier. Low average precision (less than 0.60) for A shows that it does not learn the target attributes *Y* and predicts the same label for each datapoint. On the other hand, All has high accuracy but is highly unfair. As an example, it has an odds difference of 0.38 on the Adult and 0.27 on the MEPS dataset.

On training a balanced classifier for the Adult dataset, our algorithm achieved higher accuracy than All and almost a 0 odds difference. On investigating this dataset, we noticed that the identified proxy attributes did not help with prediction, and ignoring those attributes helped with both accuracy and fairness. Some of the attributes used by our technique for classifier training after removing the proxy attributes were education and capital in Adult and purpose and age in German. In MEPS, our approach used diagnostic features like cancer diagnosis and blood pressure for prediction. We observed similar results on changing the training algorithm to random forest and AdaBoost classifier.

In addition to comparing the odds difference, we considered the causal graph for Adult and German from the prior literature [2] and used it as a ground truth to test the correctness of our algorithm. Overall, Algorithm 2 identified 95% of the proxy attributes for these datasets. In terms of running time, our presented technique was completed in less than 10 min on all datasets.

### 4.3. Synthetic Dataset

In this experiment, we considered different synthetic datasets and calculated the fraction of proxy attributes identified by Algorithm 2. Since the causal graph was used to generate data, we can verify the correctness of identified proxy attributes for these datasets. The first experiment considered causal graphs corresponding to Adult and German where the structural equations of the causal graph followed a multivariate Gaussian distribution. We used the graphical lasso variant of our algorithm for these datasets. Our algorithm identified all proxy attributes on both datasets, and none of the non-proxy attributes were labeled incorrectly.

The second experiment considered random causal graphs containing 20, 40, 60, 80, and 100 attributes consisting of 5 proxy-protected attributes, generated according to the Erdos-Renyi model where every pair of attributes was connected with probability p=0.2. In this case, Algorithm 2 achieved 100% accuracy to identify proxy attributes. To further study the effect of probability *p*, we considered higher values of p=0.5 and 0.75. In such cases, Algorithm 2 identified 83% of the proxy attributes correctly where the high degree nodes were not identified. These attributes were neither labeled as proxy nor non-proxy.

**Figure 2:**
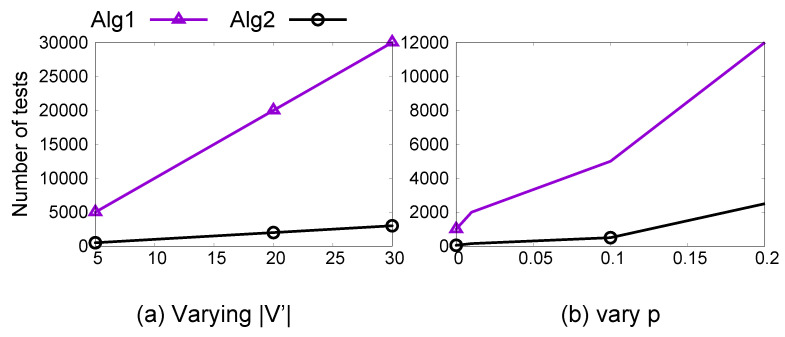
Complexity comparison of our techniques for varying dataset sizes.

**Complexity**Figure 2a shows the effect of an increase in the number of proxy attributes $V^{\prime }$ on the number of required conditional independence tests by Algorithms 1 and 2. In this experiment, we considered a causal graph of 50 attributes and varied the number of proxy attributes from 5 to 30. The complexity of both techniques increased linearly with an increase in $|V^{\prime }|$, and Algorithm 2 is orders of magnitude better than Algorithm 1. In Figure 2b, we varied the edge formation probability *p* of the generative model while keeping the size of $V^{\prime }$ constant. In this experiment, the total number of tests required increased with increasing *p*, but Algorithm 1 required much more tests as compared to Algorithm 2. This experiment validated the effectiveness of Algorithm 2 to reduce the number of CI tests required to identify proxy attributes.

In terms of running time, Algorithm 2 ran within 10 minutes for all real-world datasets. In Figure 2, its running time increased proportionally to the increase in the number of CI tests.

**Effect of feedback set size** As an additional experiment, we varied the feedback set size and evaluated the difference in results for real datasets. We observed that our approach ensures fairness whenever the feedback set contains more than 25 samples. An increase in feedback ensures that our technique is stable and ensures fairness across different runs. Whenever the number of samples is small, the behavior of our approach varies. This varied behavior is because our algorithm uses RCIT as a black-box algorithm to test conditional independence, and it returns spurious answers for small sizes of the feedback set.

Overall, this experiment validates that our technique is effective in identifying proxy attributes and mitigating unwanted biases.

## 5. Related Work

There has been very little work to consider fairness in the absence of protected attributes. Refs. [28,29] consider adversarial reweighting and empirical risk minimization techniques to learn a fair classifier in the absence of demographic information. These techniques do not assume knowledge of protected attributes, but do not study the causal impact of the unobserved features on the target attribute. Ref. [7] tackles the absence of protected attributes using transfer learning from a different dataset that does have protected attributes. Ref. [30] studies fair class balancing techniques in the absence of protected attributes. There has been some recent interest in studying the effect of noisy attributes on the fairness of classification. Ref. [31] studied the problem of training a fair classifier in the presence of noisy protected attributes. This work does not consider the causal fairness paradigm and does not directly extend to settings where the protected attribute is unobserved. Ref. [32] considered fairness in the presence of noise in the target attribute. These techniques are not directly applicable to our problem setting.

The literature on mitigating unwanted biases considers two types of fairness measures: associational and causal. Associational methods [33,34,35,36,37,38] have been shown to fail in distinguishing spurious correlations and causal dependence between attributes [3]. Identifying proxy attributes for these techniques is outside the scope of this work. There has been much recent interest in studying causal fairness frameworks [1,10,11,12,13,14,15,17,18,19,39] to achieve fairness. Ref. [2] studies the effect of different causal paths from the protected attributes on the target attribute assuming knowledge of the protected attribute and the underlying causal graph. Ref. [3] studies the problem of changing input data distribution in order to ensure interventional fairness. All these techniques require accurate characterization of the protected attribute for all data points. Extending these techniques [2,3] to leverage the information about proxy attributes in the absence of protected attributes is orthogonal to this work and an interesting question for future work.

## 6. Conclusions

In this work, we formalized a feedback based framework for interventional fairness in settings where the protected attribute is unobserved. Specifically, we examined systems where the auditors, decision makers, or affected individuals report issues in the deployed classifier. These flagged samples that suffered from biased prediction are considered indirect knowledge about the unobserved protected attributes. In this setting, we developed efficient techniques that use conditional independence (CI) testing over the observational data to formulate a constraint satisfaction problem, which identifies the proxy variables. Our techniques partition the variables into different categories based on the output of the performed CI tests. We theoretically proved the correctness of our algorithm, bound its complexity for popular causal graph models, and demonstrated its efficacy on real-world and synthetic datasets.

## Figures and Tables

**Table 1:** Notation Table.

Symbol	Meaning
*S*	Unobserved protected attribute
V	Set of attributes (also known as variables of the causal graph)
*D*	Input dataset containing V attributes
*Y*	Prediction attribute
$Y^{\prime }$	Classifier output
*F*	Feedback attribute
$D^{\prime }$	Feedback set
$V^{\prime }\subseteq V$	Proxy attributes
$V_{F}\subseteq V$	Parents of *F* in the causal graph

**Table 2:** Conditional independence properties for a pair of attributes $X_{1},X_{2}\in V$ such that $(X_{1},X_{2})\notin E$ where the output of conditional independence tests varies based on the set that $X_{1},X_{2}$ belong to and vice versa. For example, $X_{1},X_{2}\in V^{\prime }\cap V_{F}$ iff X1⊥X2|A and X1⊥X2|A,F for all $A\subseteq V\backslash \{X_{1},X_{2}\}$.

Conditions on $X_{1},X_{2}$	Conditioning on *D*	Conditioning on $D^{\prime }$
$X_{1},X_{2}\in V^{\prime }\cap V_{F}$	X1⊥X2|A for all $A\subseteq V\backslash \{X_{1},X_{2}\}$	X1⊥X2|A,F for all $A\subseteq V\backslash \{X_{1},X_{2}\}$
$X_{1},X_{2}\in V_{F}$ and ($X_{1}\notin V^{\prime }$ and/or $X_{2}\notin V^{\prime }$) (Lemma 3) and	$X_{1}⊥X_{2}|A$ for some $A\subseteq V\backslash \{X_{1},X_{2}\}$	X1⊥X2|A,F for all $A\subseteq V\backslash \{X_{1},X_{2}\}$
$X_{1},X_{2}\in V^{\prime }$ and ($X_{1}\notin V_{F}$ and/or $X_{2}\notin V_{F}$) (Lemma 2)	X1⊥X2|A for all $A\subseteq V\backslash \{X_{1},X_{2}\}$	$X_{1}⊥X_{2}|A,F$ for some $A\subseteq V\backslash \{X_{1},X_{2}\}$
$X_{1}\in V^{\prime }\backslash V_{F}$ and $X_{2}\in V_{F}\backslash V^{\prime }$	$X_{1}⊥X_{2}|A$ for some $A\subseteq V\backslash \{X_{1},X_{2}\}$	$\left(X_{1}⊥X_{2}|A,F\right)$ for some $A\subseteq V\backslash \{X_{1},X_{2}\}$
$X_{1}\notin V^{\prime }\cup V_{F}$	$X_{1}⊥X_{2}|A$ for some $A\subseteq V\backslash \{X_{1},X_{2}\}$	$\left(X_{1}⊥X_{2}|A,F\right)$ for some $A\subseteq V\backslash \{X_{1},X_{2}\}$

**Table 3:** Comparison of accuracy (Acc), average precision (AvgP), and absolute odds difference (AOD).

Dataset	OurApproach			All			A		
	**Acc**	**AvgP**	**OD**	**Acc**	**AvgP**	**OD**	**Acc**	**AvgP**	**OD**
Adult	0.79	0.78	0.025	0.80	0.75	0.06	0.75	0.47	0.03
Adult-balanced	0.78	0.71	0.068	0.65	0.59	0.38	0.63	0.59	0.40
MEPS	0.85	0.75	0.09	0.86	0.77	0.15	0.83	0.41	0
MEPS-balanced	0.77	0.67	0.25	0.77	0.67	0.27	0.76	0.59	0.05
German	0.74	0.7	0.075	0.79	0.71	0.12	0.72	0.44	0.003
German-balanced	0.70	0.66	0.06	0.72	0.67	0.13	0.6	0.53	0.05

## Data Availability

The datasets used in the study are openly available, and we provide the relevant citations.
